# Additional evidence that valence does not affect serial recall

**DOI:** 10.1177/17470218221126635

**Published:** 2022-10-18

**Authors:** Dominic Guitard, Ian Neath, Jean Saint-Aubin

**Affiliations:** 1 École de psychologie, Université de Moncton, Moncton, New Brunswick, Canada; 2 University of Missouri, Columbia, MO, USA; 3 Virginia Tech, Blacksburg, VA, USA

**Keywords:** Valence, serial recall, short-term memory

## Abstract

In immediate serial recall, a canonical short-term memory task, it is well established that performance is affected by several sublexical, lexical, and semantic factors. One factor that receives a growing interest is valence, whether a word is categorised as positive (e.g., happy) or as negative (e.g., pain). However, contradictory findings have recently emerged. Tse and Altarriba in two experiments with one set of stimuli and fixed lists concluded that valence affects serial recall performance, while Bireta et al. in three experiments with three sets of stimuli and randomised lists concluded that valence does not affect serial recall performance. Two experiments assessed the experimental discrepancy between Tse and Altarriba and Bireta et al. For both experiments, in one block, every participant saw the exact same lists as those used in Tse and Altarriba, and in the other block, each list was randomly constructed for each participant, as was done in Bireta et al. In Experiment 1, with concrete words varying in valence, we replicated the results of Tse and Altarriba with fixed lists and the results of Bireta et al. with randomised lists. In Experiment 2, with abstract words with both fixed and randomised lists, we replicate the absence of effect valence like Tse and Altarriba and Bireta et al. Overall, we conclude that valence does not affect serial recall and the discrepancy was attributed to the peculiarity of the fixed lists used by Tse and Altarriba.

Although there is a long history of research on the impact of emotion on episodic memory (Bowen et al., 2018), there are comparatively few studies examining the effects of emotion on short-term memory. In the literature, it is common to divide emotion into three components (e.g., Osgood et al., 1957). Valence is whether a word is positive (e.g., *vacation*) or negative (e.g., *pain*), arousal can be thought of as going from calm (e.g., *asleep*) to excited (e.g., *typhoon*), and dominance can be thought of as going from submissive or “has things done to it” (e.g., *meal*) to dominant or “does things to others” (e.g., *earthquake*). It has only recently been shown that arousal affects immediate serial recall when valence and dominance are equated (Landry et al., 2022), and that valence does not affect serial recall when arousal and dominance are equated (Bireta et al., 2021). Tse and Altarriba (2022) revisited the effect of valence in immediate serial recall and reported two experiments that yielded two main conclusions. The first is not controversial: Even when concrete and abstract words are equated for valence, performance on an immediate serial recall test is better for concrete than abstract words. This result replicates the many earlier demonstrations, such as those of Walker and Hulme (1999, Experiments 1 and 2), Allen and Hulme (2006, Experiment 1), Miller and Roodenrys (2009, Experiment 1, low-frequency condition), and Neath and Surprenant (2020, Experiments 1–3).

The second result, that valence affects serial recall, is the focus of this comment. In their Experiment 1, Tse and Altarriba (2022) factorially manipulated valence and concreteness. Although there was an effect of concreteness when equating for valence (i.e., the positive concrete words were recalled significantly better than the positive abstract words), there was no effect of valence when equating for concreteness. For the effect of valence on concrete words, *t*(45) = 2.008, *p* = .051, *d* = 0.296, BF_10_ = 1.000, and for the effect of valence on abstract words, *t*(45) = 1.664, *p* = .103, *d* = 0.245, BF_10_ = 0.573. In their Experiment 2, they added a neutral condition, but we focus again on the difference between positive and negative words. This time, they did observe a significant effect of valence on the concrete words, *t*(45) = 2.745, *p* = .009, *d* = 0.405, BF_10_ = 4.382, but not on the abstract words, *t*(45) = 0.762, *p* = .451, *d* = 0.112, BF_10_ = 0.210. Thus, of four comparisons, only one Bayes factor (BF) provides evidence for the idea that valence affects performance on immediate serial recall tests.

In contrast to Tse and Altarriba’s (2022, p. 10) conclusion that “word valence affected serial recall,” Bireta et al. (2021, p. 35) concluded that “there is no effect of valence on an immediate serial recall task.” This latter conclusion was based on the results of three experiments that manipulated valence while equating the words on numerous other dimensions. Moreover, each experiment used a different set of stimuli, and the BFs all indicated evidence supporting the null hypothesis (BF_01_ = 9.22, 8.76, and 9.19 for Experiments 1, 2, and 3, respectively).

We suspect the differing conclusions of Bireta et al. (2021) and Tse and Altarriba (2022) about whether valence affects serial recall are due to differences in experimental design. For simplicity, we focus on the positive concrete and negative concrete words because, as noted above, these resulted in the only significant effect of valence. These two sets of words were equated on a number of dimensions including concreteness, frequency, length, and arousal, but differed in valence.^1^ Importantly, the statistical analyses that support the statement that the words do not differ on these dimensions were done comparing the 35 words in one group to the 35 words in the other. The potential problem, as we see it, is that Tse and Altarriba then used fixed lists rather than random lists: Every participant saw the exact same lists. The reason that this is a potential problem is that the individual lists were not equated.

Here is a specific example of the type of unwanted systematic variation that can occur when the participants all see the same lists but the lists (as opposed to the set of words as a whole) were not equated. Using SUBTLEX_US_ as the measure of frequency (Brysbaert & New, 2009), we rank ordered the five positive concrete and five negative concrete lists.^2^ Four of the five highest frequency lists were positive, and four of the five lowest frequency lists were negative. Note that although overall frequency was equated, *t*(73) = 1.293, *p* = .200, the positive concrete lists nonetheless tended to be of higher frequency than the negative concrete lists because of how the words were allocated to each list. Given that high-frequency words are better recalled on immediate serial recall tests than low-frequency words, it is not clear whether the observed difference between positive concrete and negative concrete words is due to frequency or valence or some combination of the two.

Like Tse and Altarriba (2022), Bireta et al. (2021) also equated their positive and negative words by pool, but instead of having each participant see the same lists, they randomly generated each list for each participant. Randomisation minimises the chance that there is any unwanted systematic variation between the two conditions. If by chance the positive lists are higher in frequency than the negative lists, this should occur for only one participant. In contrast, if the same lists are used for each participant, then every participant experiences positive lists that are higher in frequency than the negative lists.

The suggestion that the different results of Bireta et al. (2021) and Tse and Altarriba (2022) are due to methodological differences is empirically testable. If we take the stimuli and design from Tse and Altarriba, such that each participant sees the same lists, we should replicate their results and see an advantage for positive concrete words over negative concrete words. In contrast, if we use the same stimuli but randomly construct each list for each participant, then we should minimise the likelihood of any unwanted systematic variations between the positive and negative conditions and we should replicate the null effect of valence reported by Bireta et al. (2021).

## Experiment 1

In Experiment 1, we examined whether the discrepancy between the results of Tse and Altarriba (2022) and Bireta et al. (2021) was driven by differences in experimental design. The experiment was divided into two blocks that were counterbalanced across participants. Both blocks used the same stimuli as Tse and Altarriba. In one block, every participant saw the exact same lists as was done in Tse and Altarriba, whereas in the other block, each list was randomly constructed for each participant, as was done in the work of Bireta et al. If the absence of randomisation in Tse and Altarriba’s lists was the determinant factor in causing the different results, then the effect of valence in the two blocks should differ. If the absence of randomisation is not a factor, then the effect of valence in the two blocks should be identical.

### Method

#### Sample size calculation

The Bayes factor design analysis (BFDA; Schönbrodt & Stefan, 2018) was used to estimate our sample size with a BF > 3 as the decision boundary. Analyses were conducted for the likelihood of finding evidence in favour of the alternative hypothesis and in favour of the null hypothesis. For the alternative hypothesis, the effect size of valence of Tse and Altarriba’s (2022) Experiment 2 for concrete words was used (Cohen’s *d* = 0.405). For the null hypothesis, an absence of effect was used (Cohen’s *d* = 0.000). For both sample size analyses, 10,000 simulations were conducted via a non-directional Bayesian paired sample *t*-test and the default priors.

For the alternative hypothesis simulations (Cohen’s *d* = 0.405), our results revealed that with 44 participants, 7.5% of the samples showed evidence for the null hypothesis (BF < 0.3333), 37.5% were inconclusive (0.3333 < BF < 3), and 55% showed evidence for the alternative hypothesis (BF > 3). For the null hypothesis simulation (Cohen’s *d* = 0.000), our results revealed that with 44 participants, 1.4% of the samples showed evidence for the alternative hypothesis (BF > 3), 20.5% were inconclusive (0.3333 < BF < 3), and 78.1% showed evidence for the null hypothesis (BF < 0.3333). Overall, we conclude that 44 participants would allow us to detect a similar difference to Tse and Altarriba (2022).

### Participants

The participants were recruited from Prolific (https://www.prolific.co/). All 44 participants met the following eligibility criteria: (1) native speakers of English, (2) American nationality, (3) normal or corrected-to-normal vision, (4) no cognitive impairment or dementia, (5) no language-related disorders, (6) ages between 18 and 30 years, and (7) had an approval rating of at least 90% on prior submissions at Prolific. They were paid £9.00 per hour (pro-rated) for their participation. The mean age was 25.64 years (*SD* = 3.61, range 18–30). Twenty-four self-identified as female, 19 as male, and one preferred not to specify their gender. This research was approved by the research ethics committee of the Université de Moncton.

### Materials

The stimuli were the 35 concrete negative words and the 35 concrete positive words of Tse and Altarriba (2022). In one block, their five concrete negative and five concrete positive lists were used, and in the other block, the concrete negative and concrete positive lists were randomly generated for each participant.

### Design

The experiment was a 2 valence (concrete negative words vs concrete positive words) × 2 list type (fixed lists vs randomised lists) repeated-measures factorial design. The experiment was divided into two blocks of 20 trials (10 negative trials and 10 positive trials) each corresponding to a list condition (fixed lists, randomised lists) preceded by two practice trials. The order of the blocks was counterbalanced across participants so that half of the participants began with the fixed lists and the other half with randomised lists. For both blocks, valence (negative lists or positive lists) varied randomly from trial to trial and the order of the words within a list was randomised for each participant.

### Procedure

The procedure was based on Tse and Altarriba’s (2022) study. The participants were tested in one online session lasting approximately 20 min controlled via PsyToolKit (Stoet, 2010, 2017). The experiment was self-paced by the participant and each trial was initiated by participants pressing the space bar or after the maximum delay of 60 s. Immediately after the trial was initiated, the seven to-be-remembered words were sequentially presented at a rate of one word every second with an interstimulus interval of 1 s (1,000 ms on 1,000 ms off). The words were presented on a black background in white lowercase 30 points Times New Roman font, in the centre of the computer screen. After the presentation of the last word, the message “Type the first word” was presented and participants typed the first word. Once participants entered their response by pressing the space bar, the typed word disappeared, and the message was updated, “Type the second word.” This was repeated until all seven words were typed. Participants were instructed to type the word “Skip” if they were unable to remember a word at a given serial position.

### Data analysis

The responses were scored as in the work of Tse and Altarriba (2022): A word was counted as correctly recalled only if it was recalled in the correct order.

All analyses were conducted with the statistical software *R* (R Core Team, 2021). We computed both frequentist and BF analyses, the former for descriptive information and the latter to guide our inferences. The BF analyses were computed with the “BayesFactor” R package with the default parameters (version 0.9.12–4.2; see R. Morey & Rouder, 2018; Rouder et al., 2009) and the frequentist analyses were computed with the R package “lsr” (version 0.5; Navarro, 2015). For BF analyses, we report BF_10_ corresponding to the strength of evidence in favour of the alternative hypothesis (concrete negative words ≠ concrete positive words) or BF_01_ (BF_01_ = 1/BF_10_) corresponding to the strength of evidence in favour of the null hypothesis.

### Data availability

The data and the R markdowns associated with this and the subsequent experiment are available on the Open Science Framework (OSF) project page of this project.

### Results

To allowed direct comparison between our experiment and the work of Tse and Altarriba (2022), the influence of valence on the proportion of correct responses was investigated via separate analyses for each list condition (fixed lists, randomised lists). A preliminary analysis showed no effect of block order, *F*(1,42) = 0.660, *p* = .421, $\eta_{\text{p}}^{2}$ = .015, BF_01_ = 5.276.

#### Fixed lists

**Figure 1.**
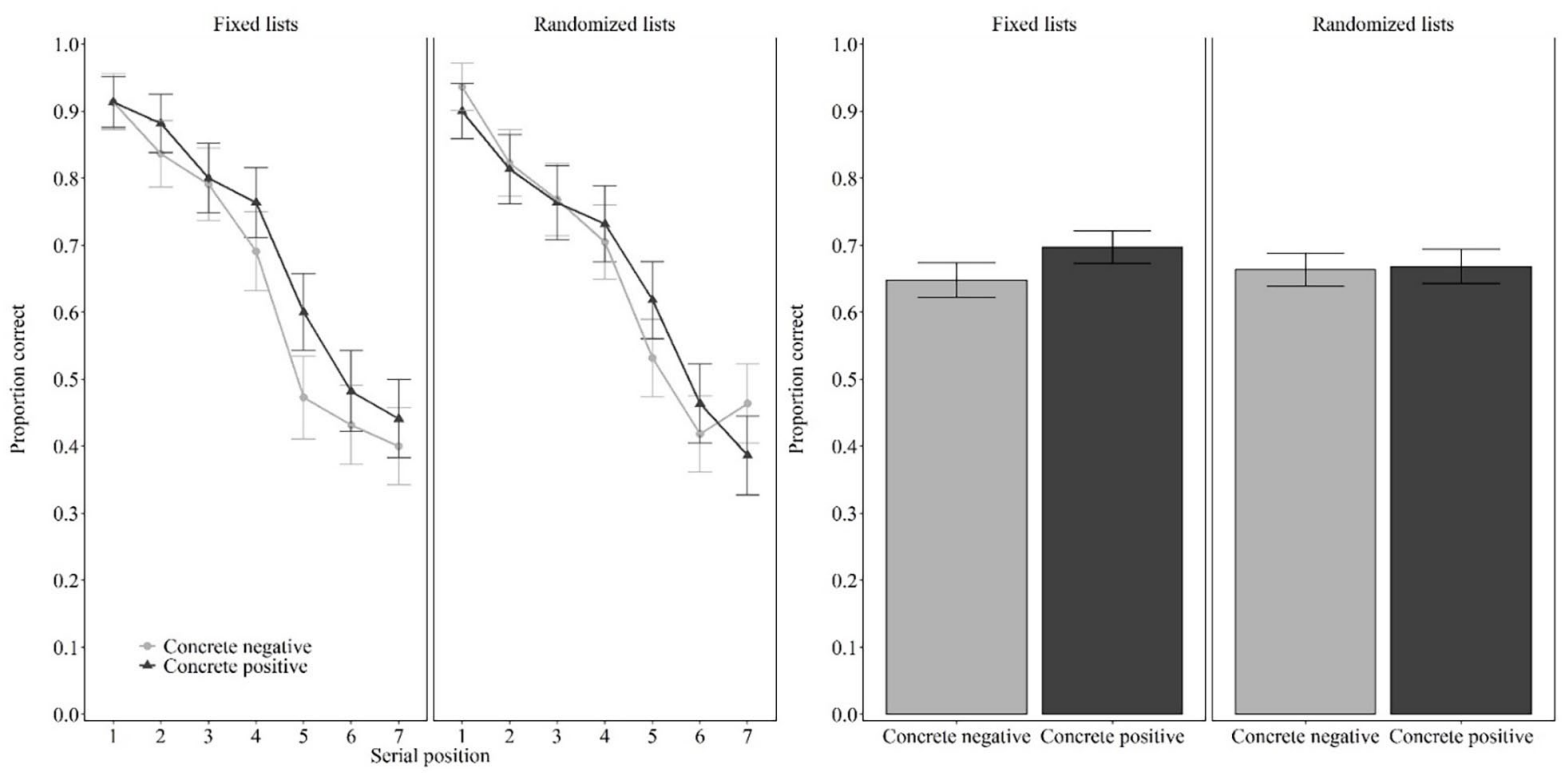
Proportion of correct response as a function of valence (concrete negative words, concrete positive words) and lists (fixed lists, randomised lists) in Experiment 1. *Note*. Left two columns, serial positions (1–7); right two columns, results averaged across serial positions. Error bars represent 95% within-participant confidence intervals computed according to Morey’s (2008) procedure.

As shown in Figure 1, when the original lists of Tse and Altarriba (2022) were used, performance was better for concrete positive words (*M* = 0.70, *SD* = 0.19) than for concrete negative words (*M* = 0.65, *SD* = 0.19). This difference was significant by the frequentist test, *t*(43) = 2.345, *p* = .024, *d* = 0.353, although the Bayesian test revealed only anecdotal evidence, BF_10_ = 1.897. This result replicates Tse and Altarriba.

#### Randomised lists

As shown in Figure 1, when using the same stimuli but with lists randomly generated for each participant, performance was the same for concrete positive words (*M* = 0.66, *SD* = 0.21) and concrete negative words (*M* = 0.67, *SD* = 0.20), *t*(43) = 0.231, *p* = .818, *d* = 0.035, BF_01_ = 5.972. This result, including the evidence in favour of the null hypothesis from the Bayesian test, replicates Bireta et al. (2021).

### Discussion

Tse and Altarriba (2022) concluded that valence affected serial recall, whereas Bireta et al. (2021) concluded the opposite, that valence has no effect on serial recall. One notable difference between the studies is the design. Experiment 1 used the stimuli of Tse and Altarriba, and compared recall when all participants saw the same lists (fixed list condition) or the lists were randomly generated for each participant (randomised list condition). Importantly, the exact same words were used in both conditions. With fixed lists, we replicated the small advantage for concrete positive words over concrete negative words reported by Tse and Altarriba, who used fixed lists. As in their study, the effect was significant with the frequentist approach, but evidence was inclusive with the Bayesian approach. With randomised lists, we replicated the null effect reported by Bireta et al., who used randomised lists. Randomised lists minimise the chance of unwanted systematic differences, whereas fixed lists guarantee that if there are any unwanted differences, they will be present for every participant.

Although these results are clear, given the theoretical importance of the absence of an effect of valence on serial recall, we conducted a second experiment using the abstract positive and abstract negative words of Tse and Altarriba (2022).

## Experiment 2

Experiment 2 was identical to Experiment 1 except that we used the abstract positive and abstract negative words of Tse and Altarriba (2022). We again compared performance when the same words were presented as fixed lists that every participant saw, as in Tse and Altarriba, or were presented in lists randomly generated for each participant, as in the work of Bireta et al. (2021).

### Method

#### Participants

Forty-four different participants were recruited from Prolific. The eligibility criteria were the same as in Experiment 1. The mean age was 25.59 years (*SD* = 3.22, range = 18–30). Twenty-seven participants self-identified as female and 17 as male.

#### Materials, design, procedure, and data analysis

The materials, design, procedure, and data analyses were identical to Experiment 1 except that we used the 35 abstract negative words and the 35 abstract positive words of Tse and Altarriba (2022). We again manipulated list type using the fixed lists from Tse and Altarriba in one block and randomly generated lists in a second block.

### Results

As in Experiment 1, there was no effect of block order *F*(1,42) = 1.543, *p* = .221, $\eta_{\text{p}}^{2}$ = .035, BF_01_ = 3.358.

#### Fixed lists

**Figure 2.**
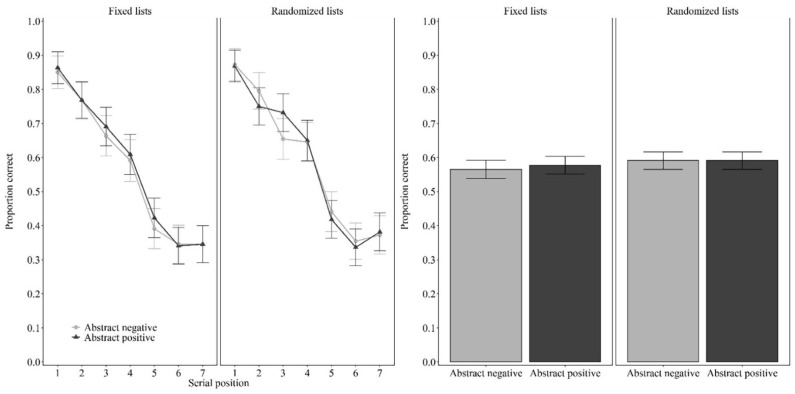
Proportion of correct response as a function of valence (abstract negative words, abstract positive words) and lists (fixed lists, randomised lists) in Experiment 2. *Note*. Left two columns, serial positions (1–7); right two columns, results averaged across serial positions. Error bars represent 95% within-participant confidence intervals computed according to Morey’s (2008) procedure.

As shown in Figure 2, when the original lists of Tse and Altarriba (2022) were used, performance was the same for abstract positive words (*M* = 0.58, *SD* = 0.23) and abstract negative words (*M* = 0.56, *SD* = 0.22), *t*(43) = –0.542, *p* = .591, *d* = 0.082, BF_01_ = 5.333.

#### Randomised lists

As shown in Figure 2, when using the same stimuli but with lists randomly generated for each participant, performance was the same for abstract positive words (*M* = 0.59, *SD* = 0.22) and abstract negative words (*M* = 0.59, *SD* = 0.21), *t*(43) = .000, *p* = 1.000, *d* = 0.000, BF_01_ = 6.126.

### Discussion

Experiment 2 was identical to Experiment 1 except for the stimuli: Whereas Experiment 1 used concrete positive and negative words, Experiment 2 used abstract positive and negative words. As in Experiment 1, there was no evidence that valence affected serial recall when each participant received randomly generated lists, replicating Bireta et al. (2021). One difference is that, whereas in Experiment 1, there was some evidence for an effect of valence when fixed lists were used, this was not replicated in Experiment 2. However, this null result replicates the null result of Tse and Altarriba. They observed an effect of valence only for the concrete and not for the abstract words.

## General discussion

As noted in the introduction, it is well known that concrete words are better recalled than abstract words on immediate serial recall tests even when the stimuli are controlled for valence (Walker & Hulme, 1999, Experiments 1 and 2; Allen & Hulme, 2006, Experiment 1; Miller & Roodenrys, 2009, Experiment 1, low-frequency condition; Neath & Surprenant, 2020, Experiments 1–3). In contrast, there are very few studies that have examined whether valence affects serial recall and even fewer that equate the positive and negative words on other dimensions. Bireta et al. (2021) created three sets of stimuli that differed in valence but were equated on other dimensions and found valence had no effect on serial recall. In particular, the BFs all indicated evidence in support of the null hypothesis. In contrast, Tse and Altarriba (2022) simultaneously manipulated concreteness and valence and did observe an effect of valence for the concrete words by a frequentist test.

The two papers used different designs. Bireta et al. (2021) randomly generated each list for each participant. Randomisation minimises the chance that there will be unwanted systematic differences between the positive and negative lists. In contrast, Tse and Altarriba (2022) used fixed lists, such that, every participant saw the same lists. This is not inherently problematic, but it can become an issue when the lists themselves are not equated. As noted earlier, it turns out that for the concrete words, four of the five highest frequency lists were positive and four of the five lowest frequency lists were negative. We tested this account by manipulating the type of list but using the same words. When Tse and Altarriba’s fixed lists were used, we replicated their results. In contrast, when randomly generated lists were used, we replicated the results of Bireta et al. Importantly, the same words were used in each type of list.

These results have important methodological implications. First, if fixed lists are used, then it is the lists that should be equated rather than the entire pool of words. If this is not done, then it is possible that one or more unwanted systematic differences may be introduced that affect the results. Second, researchers should always include their stimuli in their papers because it allows other researchers to investigate stimulus properties. The reason we know that concreteness effects obtain even when valence is controlled is because previous researchers published their stimuli and we could then compute valence using the Warriner et al. (2013) norms. Third, the data should be available for re-analysis. This enables other researchers to conduct different analyses, such as computing BFs, which were not reported in the original.

The results of the current article, along with those of Bireta et al. (2021), also have important theoretical implications. For example, the NEVER model (Bowen et al., 2018) that in general, negative words will be better remembered than positive words. The reason is that it is thought negative events lead to enhanced processing of sensory attributes relative to positive events. Although there is evidence from other tests supporting the NEVER model, the lack of an effect of valence on serial recall places limits on the model’s scope. In contrast, the current results offer some support the two-factor account of Majerus and D’Argembeau (2011). This view suggests that relative to neutral words, both positive and negative words will have enhanced item information because of their valence. Because of this, both positive and negative words will be better remembered than neutral words, but there will be no difference between positive and negative words.^3^

In conclusion, although many long-term memory/lexical factors do affect immediate serial recall, including whether the words are abstract or concrete, valence does not when the lists of positive and negative words are appropriately equated.

## Notes

1 The Warriner et al. (2013) norms include ratings for arousal, valence, and dominance. The positive concrete and negative concrete words used by Tse and Altarriba (2022) differ significantly in dominance as well as valence.

2 We used SUBTLEX_US_ rather than LogHAL because Brysbaert and New (2009) demonstrated that it accounts for a larger proportion of the variance in psycholinguistic tasks, such as lexical decision accuracy and latency than LogHAL.

3 However, it should be noted that contrary to the prediction of the two-factor account, MacMillan et al. (2022) found no difference between positive, negative, and neutral words on a recognition test.
